# DichroWeb, a website for calculating protein secondary structure from circular dichroism spectroscopic data

**DOI:** 10.1002/pro.4153

**Published:** 2021-07-21

**Authors:** Andrew J. Miles, Sergio G. Ramalli, B. A. Wallace

**Affiliations:** ^1^ Institute of Structural and Molecular Biology, Birkbeck University of London London UK

**Keywords:** bioinformatics, calculations, circular dichroism spectroscopy, data analyses, protein secondary structure, reference datasets, soluble and membrane proteins, α‐helix, β‐sheet, disordered structure

## Abstract

Circular dichroism (CD) spectroscopy is a widely‐used method for characterizing the secondary structures of proteins. The well‐established and highly used analysis website, DichroWeb (located at: http://dichroweb.cryst.bbk.ac.uk/html/home.shtml) enables the facile quantitative determination of helix, sheet, and other secondary structure contents of proteins based on their CD spectra. DichroWeb includes a range of reference datasets and algorithms, plus graphical and quantitative methods for determining the quality of the analyses produced. This article describes the current website content, usage and accessibility, as well as the many upgraded features now present in this highly popular tool that was originally created nearly two decades ago.

## 1 INTRODUCTION

Circular dichroism (CD) is a spectroscopic technique that depends on the differential absorption of left‐ and right‐circularly polarized light by a chromophore either with a chiral center, or within a chiral environment.^1^ It is regularly used in the biophysics, biochemistry, and structural biology communities to examine and quantify protein secondary structures^2^ and in the pharmaceutical industry to study higher level structures of biomolecules and to monitor changes due to environmental stresses and mutations.^3^

The far ultraviolet wavelength region (between ~260 and 190 nm) is especially useful for quantifying protein secondary structures because the signals generated are sensitive to the dihedral angles between adjacent amino acids, where stretches of similar angles in the primary chain define elements of secondary structure. The most important absorbances in this region arise from the amide chromophores through an *n* → *π* ^*^ electronic transition located at around 220 nm, and a degenerate *π* → *π* ^*^ transition centered around 195 nm. In general, the lowest wavelength that lab‐based CD instruments can accurately measure data is ~190 nm, even if experimental conditions are optimized.^4^ The accessible wavelengths can be extended into the lower wavelength vacuum UV region by using synchrotron radiation as a light source,^5^ enabling the measurement of further electron transfer transitions centered at ~175 nm^6^ and producing spectra with higher information content.

The far ultraviolet wavelength CD spectrum of a protein represents a linear sum of the spectra arising (predominantly) from the peptide bonds present in the protein, and its shapes and magnitudes are correlated with the secondary structural features of the protein (although there can also be very minor contributions [usually less than a few percent of the total magnitude] in the near ultraviolet wavelength region of the spectrum (above 240 nm) arising from aromatic or disulfide side chains.^4^). Empirical analyses are typically performed by comparing the spectrum of the query protein to spectra present in a dataset of proteins with known crystal structures,^7^ using algorithms/methods that include singular value decomposition,^8^ parameterized fits,^9^ self‐consistency,^10^ convex constraints,^11^ matrix descriptors,^12^ and neural networks.^13^, ^14^, ^15^ In all of these methods, the accuracy is generally highest for helical structures, which are composed of relatively long regular stretches of polypeptide with well‐defined dihedral angles that give rise to intense CD signals. Beta‐sheet‐rich structures tend to be more varied, due to variations in the direction and extent of their twist and the relative alignments of adjacent beta‐strands. Their spectra tend to have peak magnitudes of between one‐third and one‐fifth of those arising from helical elements.^4^, ^16^ As a result, beta‐sheet features are more complicated to identify spectroscopically, especially in mixed alpha/beta proteins, where the signals due to beta sheets can be dominated by the more intense signals due to helical elements. This bias can be somewhat mitigated if the low wavelength range of the data is extended into the vacuum ultraviolet (VUV) wavelengths below 190 nm, which can be accomplished using data collected at Synchrotron Radiation Circular Dichroism (SRCD) beamlines.^5^ This is largely because in the VUV wavelength range, β‐sheet and helical spectra tend to have opposite signs and are therefore more identifiable.^5^ Indeed, in general, data that extends to lower wavelengths provide more information so that all structural components can be more accurately determined. Other resolvable secondary structural features include turns and less common structures such as polyproline II (PPII) and 3_10_ helix,^17^ although their contributions to the overall spectrum of soluble globular proteins are minimal as they usually comprise smaller portions of the overall protein structure, and consequently the fraction of these structures present may be less accurately determined by deconvolution methods. The remaining fraction of secondary structure, which is neither helix, β‐sheet, nor turn, has variously been termed “random coil” (although it is neither random nor coil), “disordered,” or simply “other.”

Arguably the most important factor influencing the accuracy of deconvolution methods used for CD‐based secondary structure analyses (along with the wavelength range of the data) is the reference dataset used in the calculation; the accuracy of the calculation is enhanced if the proteins that comprise the reference dataset have structures similar to those in the query spectrum. Consequently, reference datasets that cover the widest ranges of secondary structure and fold space will tend to give the most accurate results.^18^ A number of publically‐available CD spectral reference datasets (covering a wide range of protein types), have been collated over the last 30 years from proteins with known (crystal) structures.^10^, ^20^, ^21^, ^22^ Some of the more recent compilations are comprised of data collected at SRCD beamlines, and thus extend the analyses to spectra containing lower wavelength data.^19^, ^20^, ^21^

## 2 THE DICHROWEB SERVER

**FIGURE 1:**
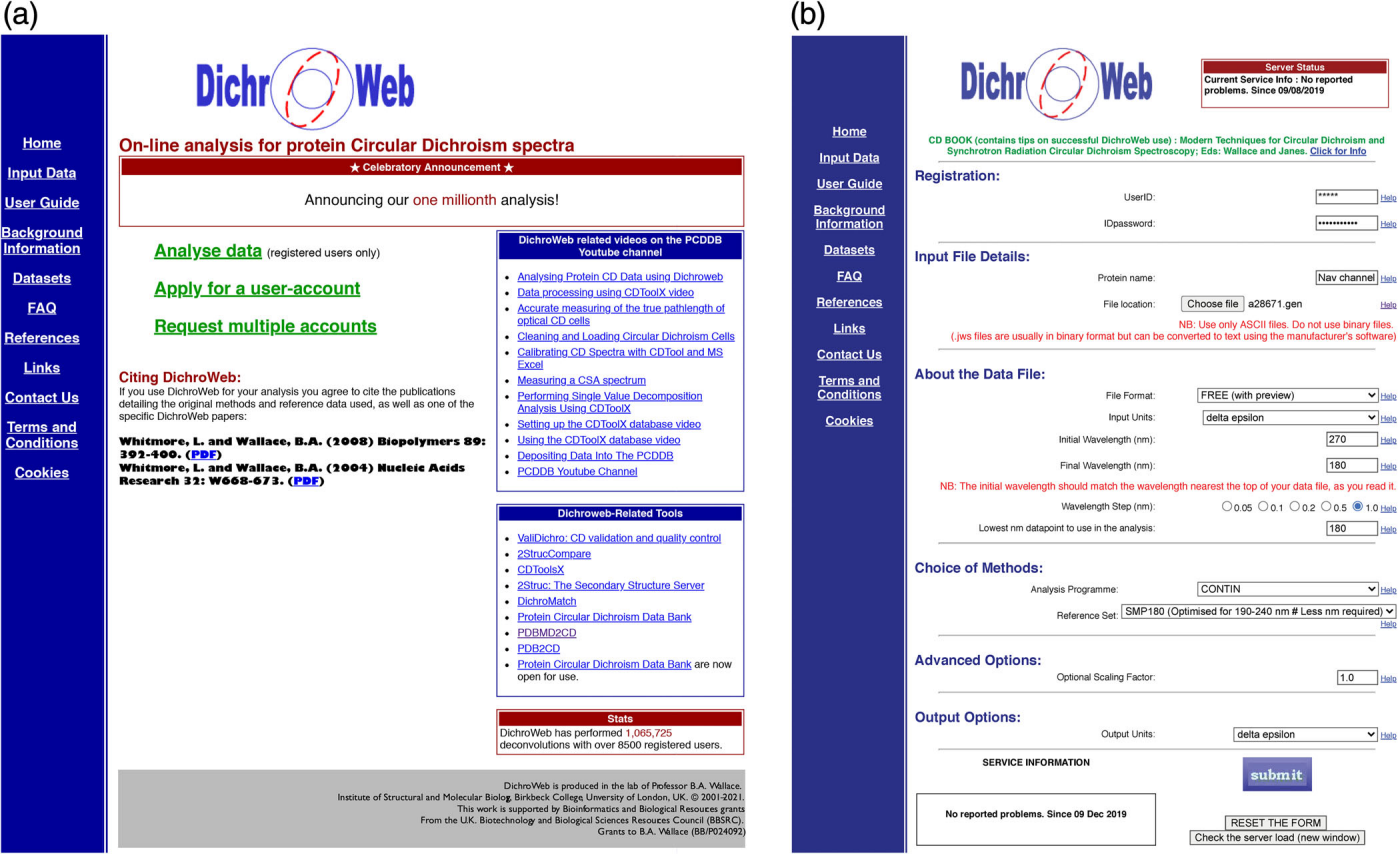
The DichroWeb Server. (a) The “Landing Page” for the DichroWeb server, located at: http://dichroweb.cryst.bbk.ac.uk/html/home.shtml. It indicates details of how to obtain an account, the link to data input/analysis sections, links to associated video guides and other related software, and usage statistics. (b) The DichroWeb server “Data Input” page

DichroWeb^22^, ^23^, ^24^ (Figure 1a) is an online server developed for the calculation of protein secondary structures from far‐UV CD and SRCD protein spectra, which provides a wide range of analysis methods and reference datasets, and produces rapid quantitative results along with visual and statistical evaluations of the analyses. Originally created in 2002,^22^ it has been updated regularly since that time with new methods, reference datasets, and graphical and tabulated means of evaluating the analysis results. This article describes the usage and tools available in the present version of this server.

When it was developed, the DichroWeb server was the first and only user‐friendly alternative to downloadable analysis software such as CDPro,^25^ which had required extensive preliminary formatting of query protein data, and provided access to limited types of reference datasets for the calculations. Downloadable software also had a disadvantage for casual users because it required the user to install, error‐correct, and ensure system compatibility with the software in order for it to function. Although a number of other online analysis servers have since been developed, that is, BeStSel,^16^ K2D2,^14^ and K2D3,^15^ DichroWeb is still the most comprehensive, well‐used, and highly‐cited resource available for CD analyses of protein structures. It provides access to a number of the most popular deconvolution algorithms (Table 1), plus 10 selectable protein spectra datasets for investigating a wide range of protein structural types (Table 2). Methods include SELCON3,^10^ CDSSTR,^8^ and VARSLC,^26^ which are based on singular value deconvolution techniques and CONTINLL,^9^, ^27^ which uses ridge regression methods. All of these methods incorporate variable selection techniques^26^ that filter the datasets so that only the closest matching spectra to the test spectrum are used in the final analysis. A neural network technique, K2D,^13^ is also available at the site. The VARSLC and K2D methods have built‐in datasets, however DichroWeb provides an extensive selection of reference sets for use with all of the other methods (Tables 1 and 2).

**TABLE 1:** Characteristics of the algorithms available in DichroWeb (and references to the original articles describing them)

Algorithm [reference]	Method	Reference datasets	Notes
SELCON3 [10]	SVD plus variable selection	1, 2, 3, 4, 5, 6, 7, 22, SP175, SMP180, SP175t	A suitable NRMSD value is ≤0.1
CONTINLL [9, 27]	RR plus variable selection	1, 2, 3, 4, 5, 6, 7, SP175, SP175t, SMP175, SMP180, SMP180t	A suitable NRMSD value is ≤0.1
CDSSTR [8]	SVD plus variable selection	1, 2, 3, 4, 5, 6, 7, SP175, SMP180, SP175t	A suitable NRMSD value is ≤0.01 Many iterations are done, so this can take longer than other methods
VARSLEC [26]	SVD plus variable selection	Built‐in dataset of 33 spectra	Requires data from 260–178 nm. No NRMSD (or other goodness‐of‐ fit parameter), nor back‐calculated spectrum produced
K2D [13]	NN	Built in weightings	Requires data from 241 to 200 nm

Abbreviations: NN, neural network; RR, ridge regression; SVD, singular value deconvolution.

**TABLE 2:** Characteristics of the reference datasets available for use in DichroWeb, and the secondary structure classifications they produce (α_R_, regular helix; α_D_, disordered (end of) helix; β_R_, regular sheet; β_D_, disordered ((end of) sheet); T, turn (any type); PP2, polyproline II; and U, unordered/other)

Reference dataset	Types of proteins in dataset	Wavelength range (nm)	Number of proteins	Structure assignments
SET1	Soluble globular	178–260	29	α_R_, α_D_, β_R_, β_D_, T, U
SET2	Soluble globular	178–260	22	α‐helix, 3_10_ helix, β, T, PP2, U
SET3	Soluble globular	185–240	37	α_R_, α_D_, β_R_, β_D_, T, U
SET4	Soluble globular	190–240	43	α_R_, α_D_, β_R_, β_D_, T, U
SET5	Soluble globular	178–260	17	helix, β, turn, PP2, U
SET6	Soluble globular and denatured proteins	185–240	42	α_R_, α_D_, β_R_, β_D_, T, U
SET7	Soluble globular and denatured proteins	190–240	48	α_R_, α_D_, β_R_, β_D_, T, U
SP175	Soluble globular	175–240	71	α_R_, α_D_, β_R_, β_D_, T, U
SP175t	Soluble globular	190–240	71	α_R_, α_D_, β_R_, β_D_, T, U
SMP180	Membrane and soluble proteins	180–240	128	α_R_, α_D_, β_R_, β_D_, T, U
SMP180t	Membrane and soluble proteins	190–240	128	α_R_, α_D_, β_R_, β_D_, T, U
Cryst175	Proteins with crystallin‐type folds	175–240	9	α_R_, α_D_, β_R_, β_D_, T, U

Of the current selectable datasets in DichroWeb, those designated 1–7, have been part of DichroWeb since its inception. They each contain between 22 (set 2) and 48 (set 7) spectra of soluble, predominantly globular, proteins. In addition, datasets 6 and 7 also include five spectra of denatured proteins, which have been found to be of use in the analyses of proteins that harbor a large amount of random coil or “other” structure. Except for K2D, all methods require experimental data which covers at least the wavelength range between 190 and 240 nm in order to contain sufficient information content for analysis of three or more types of secondary structural components. Later, two additional reference datasets, SP175,^19^ a bioinformatics‐designed dataset comprised of 71 soluble proteins, which more broadly covers fold space than any of the other reference datasets, and the specialist Cryst175,^21^ designed for the analysis of eye lens proteins from the β,γ‐crystallin family, were also made available on the site. These latter two reference datasets were produced with spectra containing SRCD data. Hence, SP175 also enables analyses for data which cover a much broader wavelength range (down to 175 nm); however, a wavelength‐truncated version of it (SP175t), can also be used with conventional CD data that only extend to 190 nm at the low wavelength end. Cryst175 is a novel reference dataset comprised of 9 spectra of proteins which have a distinctive double Greek‐key fold; it was produced because this type of fold gives rise to a unique type of spectral shape.^21^ Later, the SMP180 dataset,^20^ was created for the analysis of both membrane and soluble proteins; it contains all of the SP175 spectra plus an additional 27 soluble protein spectra and 30 membrane protein spectra, all with a low wavelength limit of 180 nm. SMP180 (and its wavelength‐truncated [at 190 nm] version SMP180t) is particularly useful because membrane protein spectra tend to differ from soluble protein spectra as these types of proteins are naturally embedded in low dielectric “solvents,” which give rise to slightly different spectral peak positions.^28^ Because of this, analyses of membrane proteins that only utilize soluble protein reference databases can be less accurate than those which include both soluble and membrane proteins.

The component spectra of the SP175 dataset proteins and their associated secondary structure and other metadata are downloadable from the Protein Circular Dichroism Data Bank (PCDDB)^29^ (https://pcddb.cryst.bbk.ac.uk/) as accession codes CD0000001000–CD0000071000. The membrane protein components of the SMP180 reference dataset are also downloadable with accession codes CD00000099000–CD00000128000; and the extra soluble proteins of the SMP180 reference dataset are downloadable with accession codes CD00000072000–CD0000098000. Table 1 lists all of the datasets currently available for each method included on the DichroWeb site, and Table 2 lists the characteristics and types of component proteins available in each of these datasets.

## 3 USAGE OF THE DICHROWEB WEBSITE

### 3.1 Data input

Data files can be directly uploaded onto the DichroWeb input page (Figure 1b). Accepted formats (Table 3) include several versions of ASCII‐formatted files generated by Jasco International Co, Ltd. (Tokyo, Japan), Aviv Biomedical Inc. (Lakewood, NJ), Olis Instruments (Athens, GA), and Applied Photophysics (Leatherhead, Surrey, UK) lab‐based instruments, a number of SRCD beamlines, and a “free” format of two columns (X,Y) representing wavelength and CD value. Data file formats produced by generic processing applications including CDTool^30^ and CDToolX^31^ are also accommodated by the “free” format. Digitized files obtained by scanning spectra from other sources such as figures in publications can be input as “BP format” files.

**TABLE 3:** Data input parameters showing the available options

Section	Input	Options available
Information about the input data and analysis parameters	File format	Free (2 column) Free (2 column) (with preview) DRS (Daresbury synchrotron format) yy (2 column) BP (bitpad scanned) (2 column) Applied Photophysics Aviv v4.1i Aviv:CDS Aviv v2.86 Jasco: v.1.30 Jasco: v1.50
	Input units	Delta epsilon Mean residue ellipticity mdeg/theta (machine units) DRS (yy units)
	Initial wavelength in the data file	First wavelength listed in data file (regardless of whether ordered from high to low, or low to high wavelengths)
	Final wavelength in the data file	Last wavelength listed in data file
	Wavelength step (interval) in the data file	1, 0.5, 0.2, 0.1 (all in nm)
	Lowest wavelength to use in analysis	(in nm) (subject to constraints of the method and database used, and quality of the data)
Choice of analysis methods	Analysis programs	SELCON3 CONTINLL VARSLC CDSSTR K2D
	Reference datasets (minimum wavelength range required)	Set1 (178–260 nm) Set2 (178–260 nm) Set3 (185–240 nm) Set4 (190–240 nm) Set5 (178–260 nm) Set6 (185–240 nm) Set7 (190–240 nm) SP175 (175–240 nm) SP175t (190–240 nm) SMP180 (180–240 nm) SMP180t (190–240 nm) CRYST175 (175–240 nm)
Advanced option	Optional scaling factor (use with caution!)	0.5–1.5×
Output options	Output units	Delta epsilon Mean Residue Ellipticity (MRE) mdeg (theta, machine units) DRS (yy units)

Users are required to manually input a number of parameters (Figure 1b, Table 3) such as the data units (i.e., mean residue ellipticity, delta epsilon [Δ*ε*], or millidegrees), the data interval, the highest and lowest wavelength collected and the lowest wavelength to use in the analysis (note that the latter may not be the lowest wavelength in the data file if the HT cut‐off value is exceeded at the lower wavelengths).^4^ Finally the algorithm and reference dataset are chosen (as discussed above), with the latter choice generally dependent upon the type of protein being studied.

An optional magnitude‐scaling factor has been added to the original version of DichroWeb so that input data can be modified by a slight amount^32^ to compensate for concentration or cell pathlength errors.^33^ The allowable scaling factors range between 0.5× and 1.5×; however, only conservative factors between ±0.1 and ±0.05 are recommended.

### 3.2 Data output

For methods with selectable reference sets, the results reflect the secondary structure fractions assigned to the proteins in the dataset used. They are, with the exception of datasets 2 and 6: alpha helix and beta sheet, both regular (R) and distorted (D), and turns, with anything else classified as “unordered” or “other.” “Distorted” helix and sheet assignments refer to the two residues at either end of a helix and the single residue either end of a stretch of sheet, which have slightly different dihedral angles from those of canonical helical and sheet structures, as defined by the Dictionary of Protein Secondary Structure (DSSP).^34^ Hence, they tend to produce slightly different CD spectra.^35^ The “Turns” component includes a combination of beta turns, bends, and bridges as defined by the DSSP.^34^ Dataset 2 and 5, include separate structural assignments of α‐helix, 3_10_ helix, β‐strand, turn, polyproline‐II helix, and unordered structures, although dataset 5 combines the two types of helix into one fraction.^25^ For the methods (VARSLC and K2D) that do not use selectable references sets, the secondary structure outputs are in terms of helix, sheet, and other (Table 2).

**FIGURE 2:**
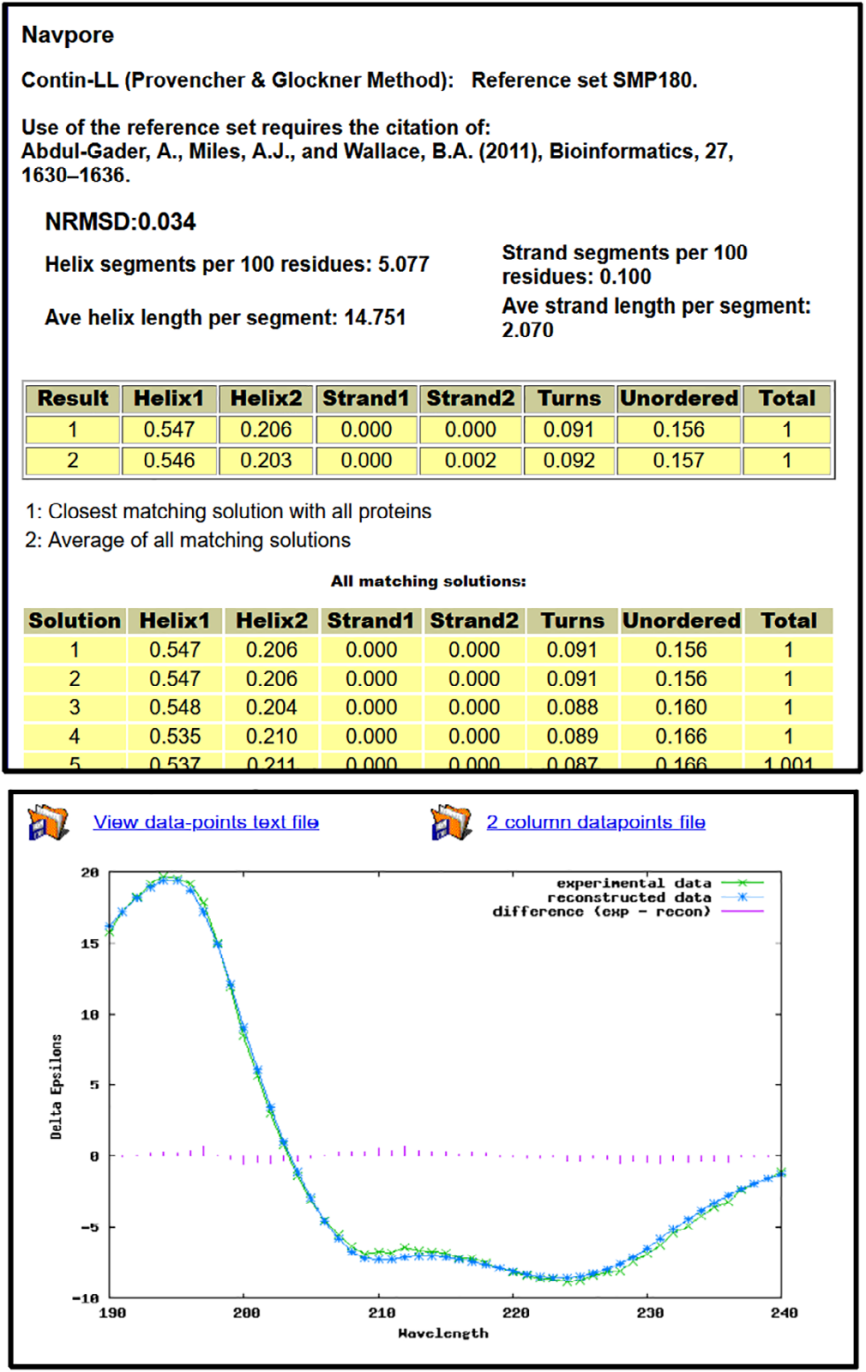
Example of DichroWeb Results Pages for a “Good” Analysis. *Top*) Example of a results page obtained using the DichroWeb server for a “good” quality analysis. The protein name [Navpore, (PCDDBid CD0006226000)] is displayed along with the analysis method used [Contin‐LL] and the reference set used [SMP180]. Below these is the NRMSD ^“^goodness‐of‐fit parameter”; the low value in this example (0.034) indicates this is a good analysis, based on the close correspondence between the back‐calculated and measured spectra. Below that is other potentially indicative information calculated for the protein (to be used [not recommended], and then only with caution). Below these are tables (in yellow overlay) which display the calculated secondary structure results obtained for (1) the closest matching solution with all proteins, and (2) the average values of all matching solutions, followed by details of all matching solutions. It is recommended that solution 1 in the top table be used, as it represents the best fit to the data. *Bottom*) Example of the graphical output of the DichroWeb server for a “good fit,” showing the experimental spectrum (green line with crosses), the back‐calculated closest match spectrum (blue line with stars), and the difference spectrum (red vertical bars) between the experimental and back‐calculated spectra. Text files for these plots can be obtained by clicking the icons at the top of the plot section

**FIGURE 3:**
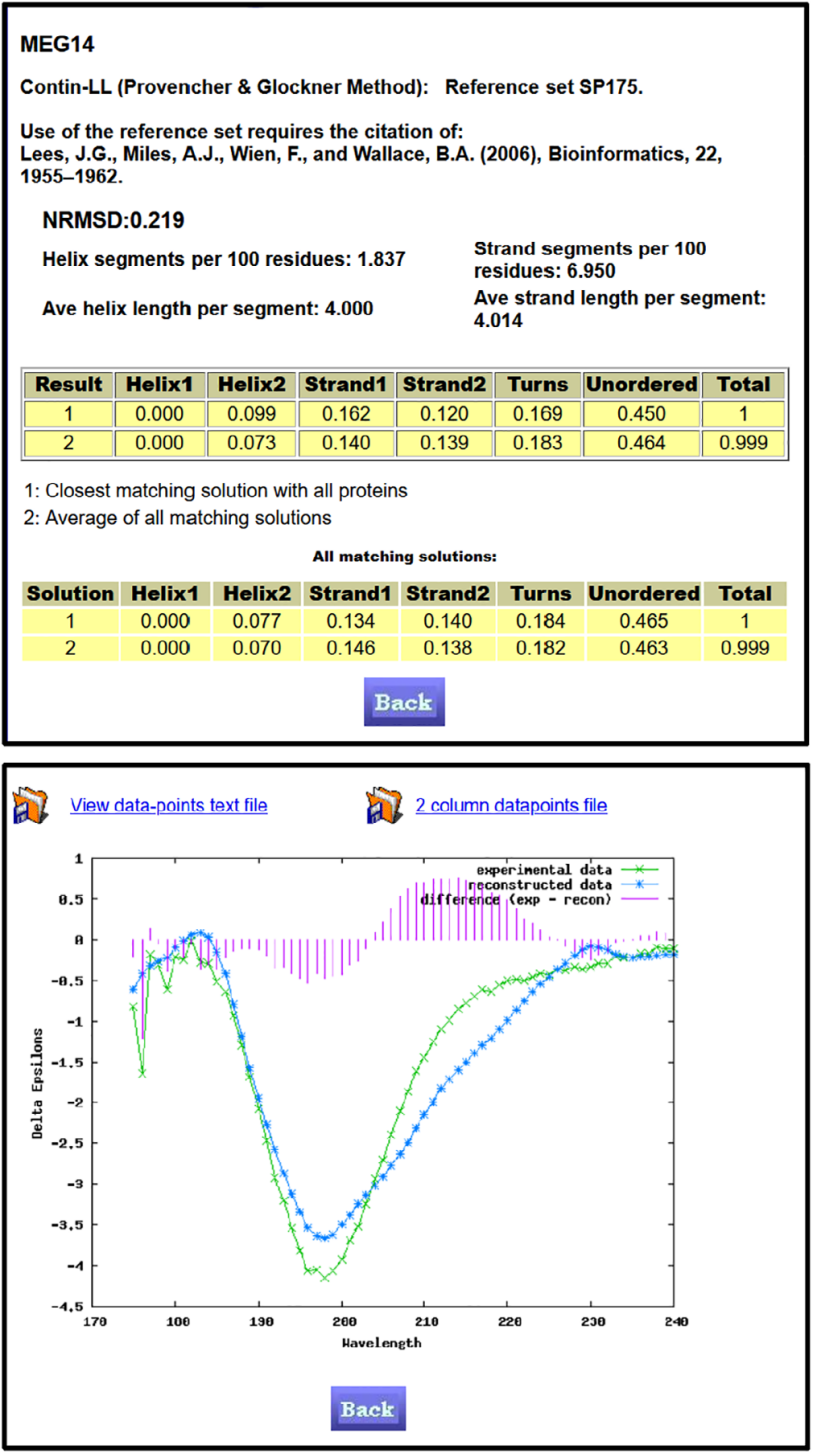
Example of DichroWeb Results Pages for a “Poor” Analysis. *Top* **)** Example of a results page obtained using the DichroWeb server for a “poor” quality analysis. This was obtained for an intrinsically disordered protein ([MEG14, (PDCDDBid CD0004055000)]**).** Features are as described in the legend to Figure 2, except in this case the high (see Table 1) NRMSD value (0.219) and the poor correspondence between the calculated and experimental spectra, suggest that the best solution is not an accurate reflection of the protein secondary structure. *Bottom*) Example of the graphical output of the DichroWeb server for a “poor fit” showing the experimental spectrum (green line with crosses), the back‐calculated closest match spectrum (blue line with stars), and the difference spectrum (red vertical bars) between the experimental and back‐calculated spectra

A plot (Figures 2 and 3, bottom) is produced showing the reconstructed spectrum of the best‐fit solution overlying the experimental spectrum, with the graphical depiction of the differences between them providing a visual means of assessing the data analysis. Figures 2 and 3, show examples of extended results tables (top) and data plots of a “good fit” and a “poor fit,” respectively. Text files (wavelength, value) of the plotted data can be downloaded.

### 3.3 Defining the quality of analyses

The “goodness‐of‐fit” parameter, included in the results table, is the Normalised Root Mean Squared Deviation (NRMSD),^36^ which provides a means of evaluating the difference between the experimental and calculated spectra. It is defined as:

$$\text{NRMSD}=\sqrt{\frac{\sum_{\lambda }{\left(\theta_{\exp}-\theta_{\mathrm{calc}}\right)}^{2}}{\sum_{\lambda }{\left(\theta_{\mathrm{calc}}\right)}^{2}}}$$

where *θ*_exp_ and *θ*_cal_ are the experimental and back‐calculated ellipticities, respectively, at each data point in the spectrum, with lower values indicating a closer match between experimental and reference data. NRMSD values tend to be higher for analyses using SELCON3 where ~0.070 would signify a good fit, lower for analyses using CONTINLL (a very good fit would be ~0.03), and lower still for CDSSTR analyses (a very good fit produces a value of ~0.007). High NRMSD values can arise from different/unusual features in the experimental spectrum compared with the standard spectra in the dataset; however, they can also indicate an error in concentration or cell pathlength measurements, resulting in an incorrect spectral magnitude and it may be useful to scale (with caution) the spectrum to compensate thereby reducing the NRMSD.^32^ Only the VARSLC algorithm does not provide a calculated spectrum or an NRMSD value.

### 3.4 User help and useful links

The comprehensive, and regularly updated, user guide in DichroWeb includes a short description (and literature citation) for each of the algorithms and a list of the proteins that comprise each selectable reference dataset. Each parameter on the input page has a link to a relevant help section. There is also a “FAQ” section that includes answers to common user queries. All of this should be considered in conjunction with the original article about the methods and datasets. Other new features include links to YouTube video tutorials on the “PCDDB Channel” (https://www.youtube.com/user/ThePcddb/videos) about various aspects of protein circular dichroism spectroscopy data collection and analyses. Included are tutorials on the use of DichroWeb, and on other related resources such as the Protein Circular Dichroism Data Bank (PCDDB),^29^ a public archive of CD and SRCD protein spectra (including all those present in the SP175 and SMP180 reference datasets).

The “user guide,” accessed from the left hand panel, provides detailed information about the input parameters and links to details about the different methods and reference datasets, and other options such as choice of wavelength range and scale factor. Additional information accessible from the left hand side bar includes: background information and references for the techniques, reference datasets and methods, the FAQ section, ways to contact the authors, and terms and conditions for use.

## 4 METRICS

DichroWeb has been used by more than 8,500 individual registered users (including more than 2,100 in the past year) from 47 countries to do >1 million analyses thus far, and has been cited (according to Google Scholar) >5,400 times. In addition, it has been used by universities for teaching classes (including many during Covid19 lockdowns for *in silico* “lab classes”), and in many international workshops and training programs over the years.

## 5 NEW FEATURES AND FUNCTIONS AND THEIR AVAILABILITY

Since it was first created in 2002^22^ DichroWeb has been continually updated with new features. These include the bioinformatics‐defined reference datasets for specialized analyses of specific protein types and protein folds.^19^, ^20^, ^21^ Many of the 12 reference datasets currently included (Table 2) specifically enable use of the more extensive wavelength range available in SRCD spectra. The protein components that comprise the reference datasets are listed in the “user guide” section (http://dichroweb.cryst.bbk.ac.uk/html/userguide_datasets.shtml). For others who might wish to develop new tools, the individual files that comprise the SP175, SMP180, and Cryst175 reference datasets are now downloadable at the Protein Circular Dichroism Data Bank.^29^

Another new feature is the scaling factor function^32^ (Table 3) which can be used when the protein concentrations (or cell pathlengths or instrument calibrations) are not precisely known.^33^ This feature was extensively tested before addition to the site, but is nevertheless to be used with caution!

To accompany the website, a number of videos have been created on the PCDDB channel of YouTube at: https://www.youtube.com/user/ThePcddb/videos, including ones describing how to process CD data, perform analyses using DichroWeb, and how to deposit and access spectra in the PCDDB.

The user interface has been updated for enhanced functionality, and a number of new options have been added, such as in the registration facility, which now includes the option for obtaining multiple passwords for teaching/workshop purposes.

In addition, it is also of note that a number of the original analysis algorithms (SELCON3, CDSSTR, CONTINLL, VARSLEC), which are included in the server, are no longer available at any other public websites, making this the only remaining online site for these methods.

## 6 FUTURE DEVELOPMENTS

In recent years there has been a growing interest in intrinsically disordered proteins (IDPs) and intrinsically‐disordered regions in proteins, which appear to be involved in important cellular processes such as control of the cell cycle, transcriptional activation, and signaling.^37^ Structural studies of IDPs are challenging since they generally do not crystallize, and therefore there are very few entries in the PDB^38^ which have large stretches or regions of disorder. Consequently, this type of protein is not represented in the current datasets that are publically available for empirical CD analyses. The spectra of largely disordered proteins tend to have a single negative peak at around 200 nm, with any small amounts of canonical regular secondary structure producing peak shoulders at higher wavelengths; intrinsically disordered regions can also produce changes to the standard peak positions and magnitudes. In some cases, their spectral profiles are similar to those generated by highly twisted β‐sheets^16^ and hence analyses using existing datasets tend to assign significant amounts of β‐sheet structure to spectra of intrinsically‐disordered proteins or proteins with intrinsically‐disordered regions. Similarly, proteins containing regions of polyproline‐like structures,^17^ often tend to have similar spectral characteristics to IDPs. At present, only DichroWeb datasets 6 and 7, which each contain five spectra of denatured proteins (which may or may not be disordered in the same way as IDPs) specifically contain this type of structure.^39^ Although those datasets do perform slightly better than the others for analyzing largely disordered spectra, any future dataset created which includes spectra of proteins with significant fractions (>50%) of disordered structures would obviously give improved results. The difficulty of creating such a dataset lies in obtaining consistent independent secondary structural assignments for the IDPs, which are mostly derived from NMR studies (where multiple conformations are in equilibrium) and bioinformatics predictions, since they are not available in crystal structures. This endeavor is currently in progress, and a bespoke dataset for analyzing IDPs is expected to be included in DichroWeb in the future.

## 7 CONCLUSIONS

DichroWeb is a user‐friendly website for calculating protein secondary structures from CD and SRCD spectroscopic data. It includes the use of a number of different algorithms and reference datasets, which make it a generic tool for examining proteins comprised of many different secondary and tertiary structural types. It has been used in a wide range of structural biology studies, with more than 1 million analyses undertaken thus far. Although it has been, and currently is, the most widely‐cited tool in use for analyzing CD spectra, a number of other specialist web‐based resources such as BeStSel,^16^ K2D2,^14^ and K2D3^15^ have emerged which enable some of the functions of DichroWeb by using different computational methods or focus primarily on specific types of protein structures. However at present (and for nearly the past two decades), DichroWeb is and has been the most comprehensive resource available for CD analyses of proteins. To facilitate usage, the DichroWeb website accepts a wide range of data formats, measurement units, data collection intervals, and wavelength ranges enabling it to be used with data from both conventional lab‐based CD instruments as well as for data collected at SRCD beamlines.

DichroWeb displays results in both table and graphic formats, and includes in the output, a goodness‐of‐fit parameter (NRMSD) indicating the correspondence between the experimental input spectrum and the back‐calculated best‐fit spectrum derived from the analysis, along with a visual comparison of the experimental and back‐calculated spectra. Additionally, the DichroWeb website provides a hub for access to extensive help and tutorial material including YouTube videos on many aspects of protein CD spectroscopic data collection, processing, and analyses.

## CONFLICT OF INTEREST

The authors declare no conflict of interest.

## AUTHOR CONTRIBUTIONS

**Andrew Miles:** Conceptualization; methodology; validation; writing‐original draft; writing‐review & editing. **Sergio Ramalli:** Conceptualization; data curation; software; writing‐review & editing. **B A Wallace:** Conceptualization; funding acquisition; methodology; resources; supervision; writing‐original draft; writing‐review & editing.

## Data Availability

This website is freely accessible online at: http://dichroweb.cryst.bbk.ac.uk/html/home.shtml. Usage for analyses requires individual users to register for accounts by application at: http://dichroweb.cryst.bbk.ac.uk/html/apply.shtml. No password is required to access the informational content. Instructors who wish to use it for teaching and training purposes can apply for group registrations at: http://dichroweb.cryst.bbk.ac.uk/html/apply‐teacher.shtml. Informational and instructional videos about DichroWeb and related CD topics can be accessed via the PCDDB YouTube channel (located at: https://www.youtube.com/user/ThePcddb/videos) including a bespoke video about DichroWeb at: https://www.youtube.com/watch?v=QZat_Wr2NGM&t=3s.
